# Living together in biofilms: the microbial cell factory and its biotechnological implications

**DOI:** 10.1186/s12934-016-0569-5

**Published:** 2016-10-01

**Authors:** Mercedes Berlanga, Ricardo Guerrero

**Affiliations:** 1 Section Microbiology, Department of Biology, Health and Environment, Faculty of Pharmacy and Food Sciences, University of Barcelona, Av. Joan XXIII, s/n, 08028 Barcelona, Spain; 2 Laboratory of Molecular Microbiology and Antimicrobials, Department of Pathology and Experimental Therapeutics, Faculty of Medicine, University of Barcelona-IDIBELL, Barcelona, Spain; 3 Barcelona Knowledge Hub, Academia Europaea, Barcelona, Spain

**Keywords:** Biofilms as cell factories, Living together, Surfactants, Protein BlsA, Synthetic microbial communities

## Abstract

In nature, bacteria alternate between two modes of growth: a unicellular life phase, in which the cells are free-swimming (planktonic), and a multicellular life phase, in which the cells are sessile and live in a biofilm, that can be defined as surface-associated microbial heterogeneous structures comprising different populations of microorganisms surrounded by a self-produced matrix that allows their attachment to inert or organic surfaces. While a unicellular life phase allows for bacterial dispersion and the colonization of new environments, biofilms allow sessile cells to live in a coordinated, more permanent manner that favors their proliferation. In this alternating cycle, bacteria accomplish two physiological transitions via differential gene expression: (i) from planktonic cells to sessile cells within a biofilm, and (ii) from sessile to detached, newly planktonic cells. Many of the innate characteristics of biofilm bacteria are of biotechnological interest, such as the synthesis of valuable compounds (e.g., surfactants, ethanol) and the enhancement/processing of certain foods (e.g., table olives). Understanding the ecology of biofilm formation will allow the design of systems that will facilitate making products of interest and improve their yields.

## Background

Our perception of bacteria as unicellular life forms can be attributed to the axenic (“pure”) culture paradigm. While suspensions of bacteria growing in liquid medium have enabled the discovery of the main features of microbial physiology and genetics, in nature bacteria rarely grow as axenic planktonic cultures. Instead, they predominantly exist as communities of sessile cells that develop as biofilms [1–3]. The term “biofilm,” coined by Bill Costerton in 1978, refers to heterogeneous structures comprising different populations of microorganisms surrounded by a matrix (mostly of exopolysaccharides) that allows their attachment to inert (e.g., rocks, glass, plastic) or organic (e.g., skin, cuticle, mucosa) surfaces [4]. Although most natural or environmental biofilms are highly diverse multi microbial communities, the basic biology of biofilm development has been studied using single-species biofilms [5].

Biofilm formation is a nearly universal bacterial trait and has several general characteristics. Thus, biofilm development can be divided into three distinct stages: attachment, maturation (active sessile cells), and release [6, 7]. In relation with the properties of the surfaces, the factors of a surface that determine initial bacterial attachment are its charge, hydrophobicity, and roughness [8].

Biofilms do not grow forever: rather, the release of previously sessile cells is an intrinsic part of the surface-associated mode of life and it leads to the formation of new biofilms, often at distant sites [9]. Bacteria are released from biofilms via desorption, detachment, and dispersion. Desorption is the direct transfer of bacteria from the biofilm surface to the surrounding medium; it usually occurs in the early stages of biofilm development. Detachment involves external forces, such as abrasion, grazing, and erosion that are sufficient to disrupt the biofilm’s structure. In dispersion, regulatory systems enable physiological changes that facilitate the release of cells from the biofilm to the medium [9]. Thus, while desorption and attachment are passive forms of “escape,” dispersion is an active process [9].

Biofilms offer bacteria several ecological and physiological advantages: Biofilms constitute a protective physical barrier to nonspecific and specific host defenses during infection; they confer tolerance to antimicrobial agents (disinfectants and antibiotics) by reducing diffusion of those toxic compounds; and they effectively reduce the grazing by protozoa [10–13]. Those protective benefits of biofilms depend on their inherent structure (matrix), and on the gene expression patterns of sessile cells [12, 14].

The structure and composition (up to 97 % water) of the biofilm matrix protect cells from desiccation [15]. By providing a stable physical environment for cell to cell contact (conjugation) or the incorporation of external DNA (transformation), biofilms facilitate horizontal gene transfer among the large number of individuals residing within them [12, 16–18].

## The benefits of living together

The structural integrity of a biofilm depends upon the extracellular matrix (ECM) produced by its constituent microorganisms. The ECM of bacterial biofilms is a complex mixture of exopolysaccharides, nucleic acids, proteins, and other compounds. Indeed the composition of the ECM may be as diverse as that of the biofilms themselves, and it contributes significantly to the organization of the community [3, 19]. Microorganisms in biofilms are metabolically and functionally integrated consortia that can adopt specific spatial configurations; the presence and localization of the different cell types is therefore dynamic [20]. The consumption by these different cell types of resources (e.g., H_2_, H_2_S, NH_3_, several organic compounds), electron acceptors (such as O_2_, SO_4_^−2^, NO_3_^−^, CO_2_, etc.), waste products, and other substances generated by microorganisms in the biofilm establishes the driving forces that lead to the formation of the chemical gradients that allow molecular diffusion [21–23].

Microbial consortia have played important roles throughout the history of life on Earth, from the microbial mats (a type of biofilm) that were probably the first ecosystems in the early Archean (about 3850–3500 million years ago), to the complex microbiota of the intestinal tract of different animals [21, 24]. Cell to cell communication is ubiquitously employed by individual microorganisms as well as microbial communities to coordinate different physiological processes and to initiate cooperative activities that depend on the production and secretion of small diffusible auto inducers (quorum sensing signals), such as acyl-homoserine lactones, and oligopeptides [25]. Interactions mediated by inducers form the basis of quorum-sensing, which governs many important physiological processes, such as biofilm development (attachment-maturation-detachment), biodegradation of pollutants, changes in virulence, and regulation of metabolic pathways (e.g., antibiotic production, exopolysaccharide secretion, and biosurfactant biosynthesis) [25, 26].

From the human perspective, microbial biofilms can be detrimental or beneficial. Biofilms hinder industrial processes by causing biofouling, reducing heat transfer, and increasing corrosion. In addition, because they often contain pathogenic and spoilage bacteria resistant to cleaning and disinfecting agents, they pose a risk to public health and compromise the quality of food and non-food products [27, 28]. In the medical setting, biofilms cause infections, especially within implants, in the urinary tract and periodontal tissue, and may complicate diseases, such as cystic fibrosis [29, 30]. The difficulty in eradicating these infections reflects the antimicrobial tolerance of bacteria protected within biofilms. In fact, the antimicrobial resistance of biofilm bacteria is 100- to 1000-fold higher than that of planktonic cells [31].

In this review, however, we focus on the beneficial applications of biofilms in the biotechnological production of organic compounds and the modification of several foods. We also consider the possible use of artificially engineered biofilms with increased capabilities designed to yield value-added products.

## Phenotypic transition of free-living cells to attached cells and to detached cells

**Fig. 1:**
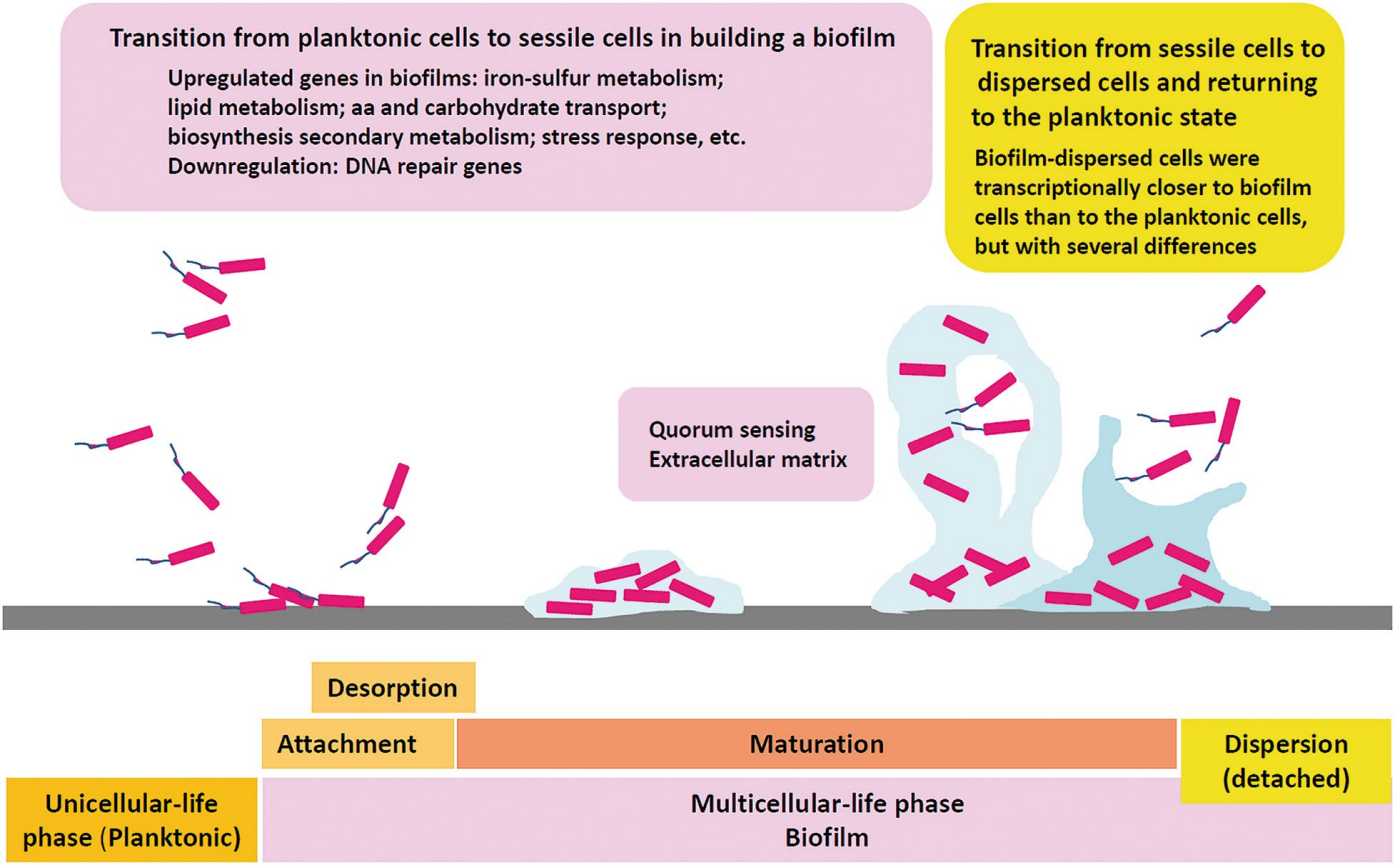
The bacterial life cycle. Unicellular (planktonic or free-swimming) and multicellular (biofilm or sessile cells) life phases alternate over time. In this two-phase cycle, bacteria undergo physiological transitions from planktonic cells to sessile cells in building a biofilm, and from sessile cells to dispersed cells in returning to the planktonic state. Each phase is associated with a unique transcriptional behavior. (Sketch by M Berlanga)

The bacterial life cycle can be divided into two distinct life phases: unicellular (planktonic) and multicellular (biofilm or sessile cells) [22]. Alternation between the two phases requires the transition from planktonic cells to sessile cells to initiate biofilm formation and from sessile cells to detached cells to allow a return to the planktonic state [32] (Fig. 1).

Comparisons of the different whole transcriptomes and/or metabolomes obtained in *Klebsiella* [33], *Acinetobacter* [34], *Haemophilus* [35], *Listeria* [36], and *Streptococcus* [37] have shown that each life phase is associated with a unique transcriptional behavior. The differences in gene expression between planktonic cells and biofilm communities include the up-regulation and down-regulation of distinct sets of genes [38]. For example, genes involved in iron-sulfur metabolism, lipid metabolism, amino acid and carbohydrate transport, biosynthesis of secondary metabolites, and stress response are up-regulated during biofilm formation [33, 38], as are genes encoding efflux system components [34]. In the case of iron metabolism, the iron concentration in the medium is an important environmental signal that induces the expression of adhesion factors, which are critical to the attachment stage of biofilm development. Accordingly, several genes involved in iron acquisition are over-expressed in the biofilm compared to planktonic cells. The down-regulation of DNA repair genes in biofilm cells indicates that the frequency of spontaneous mutations, and therefore of novel genetic traits, is elevated within the biofilm [38].

Cells dispersed from biofilms are transcriptionally closer to their parent cells than to planktonic cells and display specific phenotypes with a high adaptive ability allowing the colonization of new environments [33]. However, biofilm cells and newly dispersed cells also differ, for example, in their relative expression of genes involved in the SOS response, which are overexpressed in cells of the planktonic growth mode [33, 38].

## Using the unique features of biofilms in biotechnological applications

As noted above, the ECM of bacterial biofilms is a complex mixture of exopolysaccharides, nucleic acids, proteins and other compounds that mediate surface adhesion, cell to cell communication, self-organization within the biofilm, structural integrity, nutrient acquisition, and the antibiotic resistance of the bacterial community. Some of the compounds present in the biofilm ECM may be of biotechnological utility for, among others, the cosmetics, food, and pharmaceutical industries. In the following text we examine the potential applications of biofilm surfactants (rhamnolipids) and the biofilm protein BslA.

Microbial surfactants are surface-active metabolites that reduce surface and interfacial tension [39]. They are produced by microorganisms growing on a variety of substrates and have a diverse group of chemical structures, including glycolipids, lipopeptides and lipoproteins, fatty acids, neutral lipids, and phospholipids, in the form of polymers and particles [40]. Surfactants participate in several key biological functions in different microorganisms, such as substrate uptake [41], modification of the microbial cell surface [41], cell motility [41], and biofilm development [42–45].

Among the better-studied biosurfactants are rhamnolipids, produced mainly by *Pseudomonas aeruginosa*. In this species, rhamnolipids play an essential role in the different stages of biofilm development and therefore in the establishment of the biofilm phase of life [46]. Rhamnolipids are extracellular secondary metabolites with surface-active properties under the control of two interrelated quorum-sensing systems: *las* and *rhl* [47]. Low concentrations of rhamnolipids alter cell-surface properties by increasing the hydrophobicity of the cell, which increases its surface affinity and therefore its initial surface adherence [40, 48, 49]. However, the overproduction of rhamnolipids inhibits biofilm formation, blocks cellular aggregation, and diminishes secondary colonization onto preformed biofilms by planktonic bacteria [43]. After adhesion, low concentrations of rhamnolipids are sufficient to facilitate the aggregation of *P. aeruginosa* and therefore the initial formation of a microcolony [50]. Later on, in mushroom-shaped mature biofilms, rhamnolipid biosurfactants maintain fluid channels in an open state and thereby support the biofilm itself, by ensuring the flow of nutrients and oxygen into the community and the efflux of waste products [43]. Davey et al. [43] reported that mutants defective in rhamnolipid production were unable to maintain water channels. Finally, the active process of dispersion, in which cell detachment occurs during the late stages of biofilm formation, is also actively mediated by rhamnolipids [51].

Biosurfactants have been the subject of increasing attention because of their lower toxicity and higher biodegradability compared to their synthetic chemical counterparts [47, 52]. Despite their many commercial applications (e.g., as agents for emulsion, wetting, foaming, phase dispersion), their large-scale production has not been possible because of the low yields and high production costs [39]. Better knowledge of the genetics and regulatory pathways underlying surfactant expression is needed to improve the production of biosurfactants [47].

**Fig. 2:**
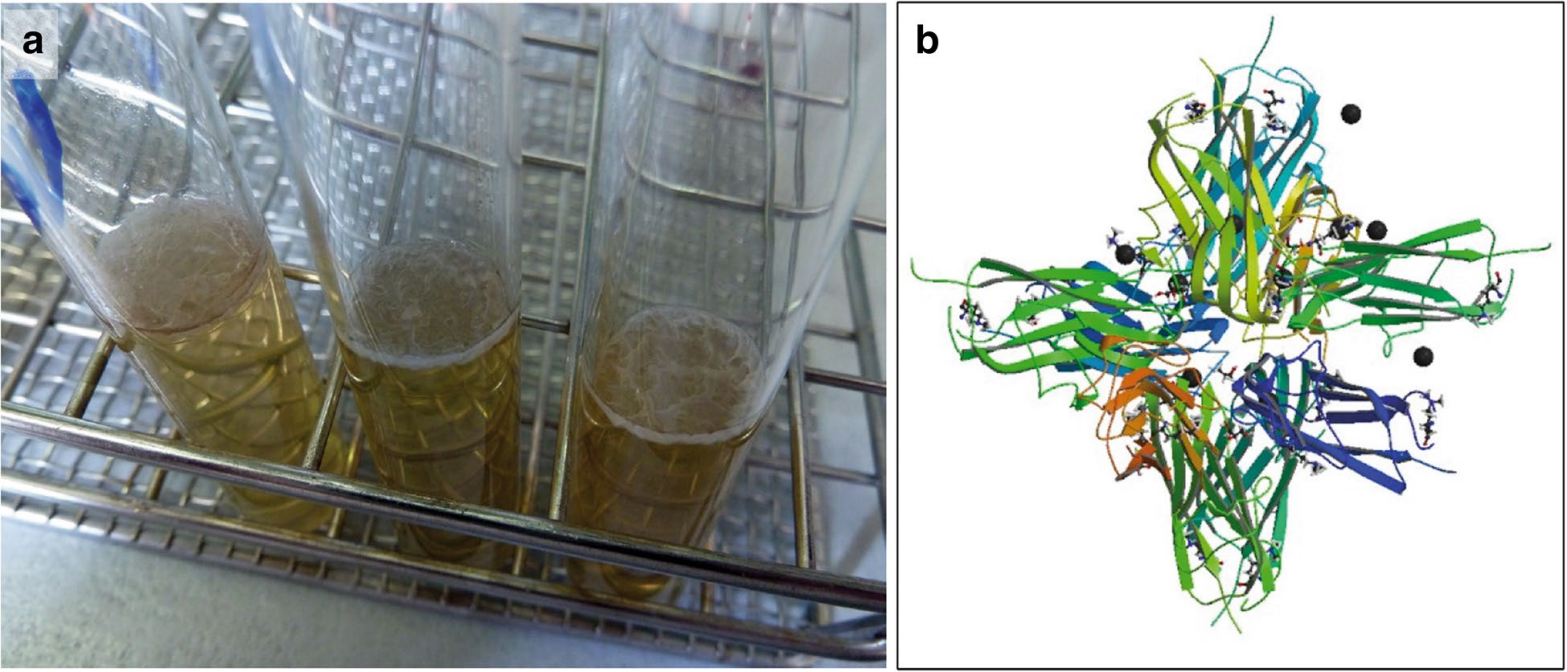
**a** Top-down view of a pellicle grown by three strains of *Bacillus* sp. incubated for 2 days at 30 °C (Photograph by M Berlanga). **b** Molecular structure of protein BslA, a bacterial hydrophobin (from the Protein Data Bank; http://www.rcsb.org/pdb/explore/explore.do?structureId=4BHU; doi: 10.2210/pdb4bhu/pdb) (Hobley et al. [53])

In the laboratory, *Bacillus subtilis* forms complex colonies on the surface of agar plates and floating biofilms (pellicles) at the air/liquid interface [53, 54]. The persistent resistance to liquid wetting and gas penetration of *B. subtilis* biofilms is probably due to the presence of the surface-active protein BslA in the biofilm matrix. In vivo, amphiphilic BslA localizes to the biofilm surface [55]. BslA is a member of the family of hydrophobin proteins and its core is structurally similar to that of proteins of the immunoglobulin superfamily [53] (Fig. 2). BslA is important for proper biofilm development, but unlike exopolysaccharides and the amyloid protein TasA it is not directly involved in cell cluster formation [56]. Moreover, it is synthesized only after the production of exopolysaccharide and amyloid fibers. The disruption of BslA production results in the loss of surface repellency and alters the surface microstructure of the biofilm [56].

Purified BslA is soluble in aqueous solution, but in air/water, oil/water or solid interfaces it forms a viscoelastic interfacial proteinaceous film [57]. Hydrophobins such as BslA may have applications in the food or cosmetic industry, as stabilizers. For example, ice cream is a mixture of air, fat, milk proteins, sugar, and water. During ice cream production, BslA protein can be used to combine the air, fat, and water, thus yielding a stable mixture and allowing ice cream to stay frozen for longer periods of time, even in hot weather. BslA also retards the growth of ice crystals, ensuring that a smooth texture is maintained [58].

## Biofilms as cell factories

Biofilms could also be used for the production of various chemicals, whether by fermentation (ethanol, butanol, lactic acid, and succinic acid) or during wastewater treatment or bioremediation. Thus far, the biofilms used to obtain industrial products are typically those of single species, which allows the controlled growth conditions needed to maximize the production of the desired compound [59–61].

In biofilms intended for industrial applications (biofilm reactors), microbial cells are fixed on different supports by adsorption, entrapment, or covalent bond formation. Adsorption uses the natural ability of bacterial cells to adhere to a support (such as charcoal, resin, vermiculite, sand particles, polypropylene). Entrapment consists of active cell immobilization in a polymer matrix (such as agar, alginate, polyacrylamide, chitosan, gelatin, collagen), whereas a prerequisite for covalent binding to surfaces is the presence of coupling agents that promote adhesion to the support [61]. Generally, the most commonly used biofilm bioreactors are fixed by adsorption, as is the case in continuous stirred tank reactors, packed bed reactors, fluidized bed reactors, and airlift reactors [59, 62–64]. In all of these, an appropriate reactor design and the correct solid support are essential to achieve homogeneous distribution of the biofilm and therefore enhance a stable production in the biofilm reactor [61]. Table 1 compares the production by adsorption-fixed biofilm reactors vs. planktonically growing cells of several compounds and shows the higher production rates of biofilms reactors. Indeed, the advantages of biofilm reactors include their ability to retain 5 to 10 times more biomass per unit volume of reactor, thereby increasing production rates, reducing the risk of cell washout at high dilution rates during continuous fermentation, and eliminating the need for re-inoculation during repeated-batch fermentation [59, 61]. Additionally, biofilms provide a stable environment for the microorganisms enclosed within them, and their ECMs confer a higher resistance to extreme conditions of pH and temperature and the presence of toxic substances [61, 62].

**Table 1:** Comparison of the synthesis of products by biofilm reactors (adhesion to different supports) vs. planktonically growing cells

Species	Product synthesized	Productivity of the biofilm (g/l/h)	Productivity of planktonically growing cells (g/l/h)	Reference
*Zymomonas mobilis*	Ethanol	105	<4	[113]
*Zymomonas mobilis*	Ethanol	536	5	[114]
*Zymomonas mobilis*	Ethanol	13.40	0.43	[115]
*Saccharomyces cerevisiae*	Ethanol	76	5	[114]
*Clostridium acetobutylicum*	Butanol	1.53	~0.22	[116]
*Actinobacillus succinogenes*	Succinic acid	8.8	7.0	[117]

In contrast to natural or laboratory-produced biofilms (obtained by adhesion to carrier surfaces) or reactor biofilms (obtained by adsorption to a support), cells immobilized or entrapped, e.g., on alginate or agar, do not undergo an adhesion step. Consequently, the changes in gene expression that normally follow adhesion are absent [33, 65].

**Fig. 3:**
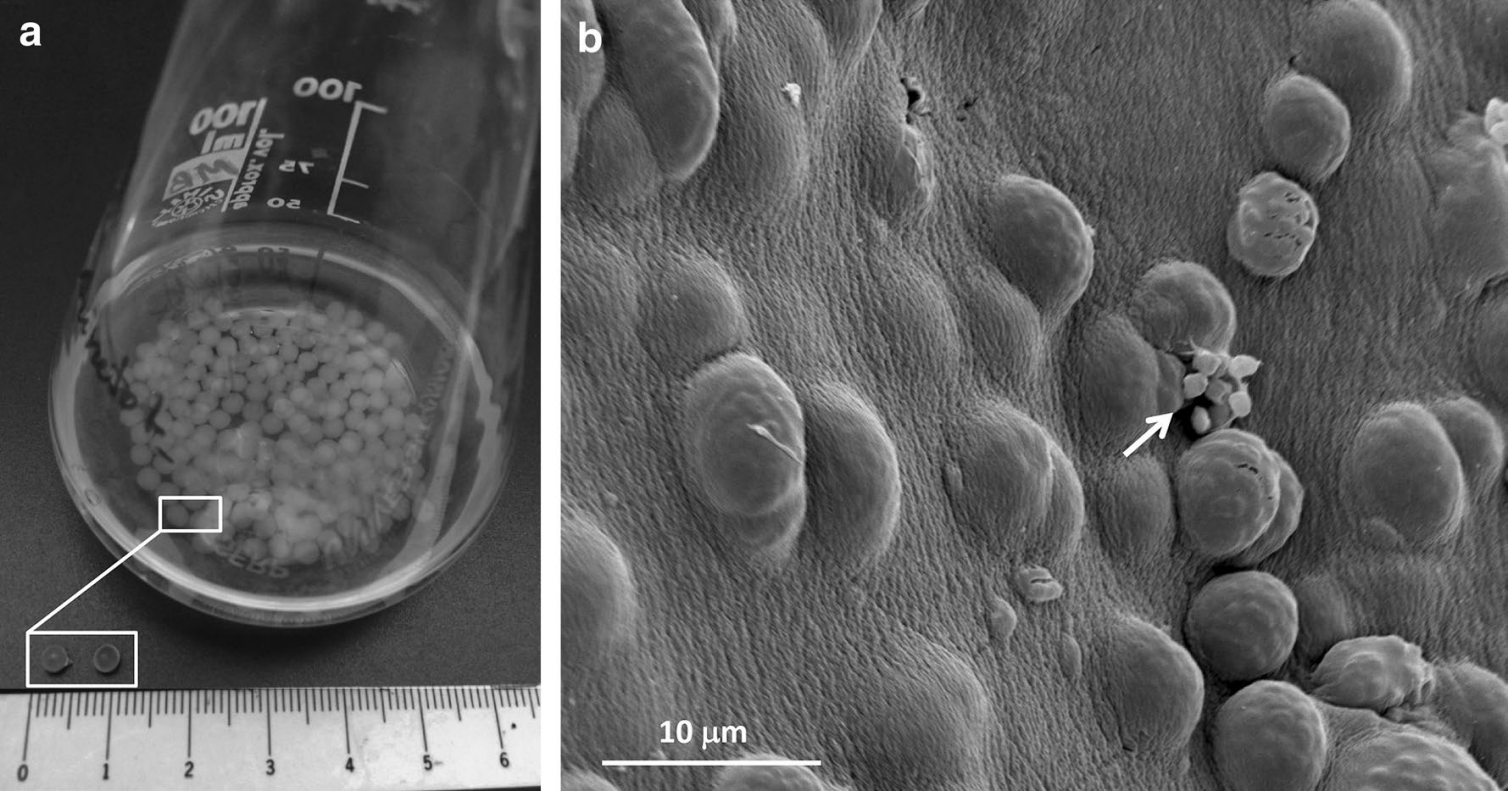
**a** Alginate beads (2 mm in diameter, see highlighted rectangle) in an erlenmeyer flask containing 50 ml of tryptic soy broth diluted 30-fold. (Photograph by M Berlanga) **b** Scanning electron micrograph of *Halomonas* immobilized cells after 24 h of incubation. (From [74], with permission). *Arrow* individual cell protruding from the bumps produced by the presence of microcolonies on the surface of the bead and about to detach

The immobilization of cells in alginate beads, could be used as a model for artificial biofilms, for several reasons: (i) protein expression: expression patterns in artificially immobilized microorganisms support the existence of a specific behavior in immobilized cells, as it is observed on “authentic” biofilms [66, 67]. (ii) Porous matrix and gradient formation: cell distribution in the beads depends on bead formation, but generally there was a greater presence of cells on the surfaces of the alginate beads than in their cores. The porosity of the beads is related to the type and concentration of the alginate. Therefore, cells located at the center of the beads may face aeration and nutritional limitations [68]. (iii) Detached cells: expanding colonies near the surface of the gel will eventually touch the gel surface and pieces of biomass could be released. In beads, it seems that dispersed cells are an eruption of entire microcolonies at once into the surrounding medium [66, 69–71] (Fig. 3). (iv) Phenotypical characteristics of the detached cells: Detached or dispersed cells from “authentic” biofilm exhibited an enhanced ability to adhere to endothelial cells [72] and other surfaces [73], similarly as it has been observed in dispersed cells from the alginate beads [74]. In *Halomonas venusta*, the surface properties (Lewis-acid or electron acceptor–donator character of the cells and their hydrophobicity) of the dispersed cells clearly differ from those of planktonic cells, and in consequence they may explain their better adhesion on polystyrene [74].

Cells immobilized on alginate beads have been used in the degradation and biotransformation of pollutants [75], the preservation of cell viability [76], and the production of enzymes [77, 78], probiotics [79] and other value products such as poly-β-hydroxyalkanoates (PHA) [69]. Dispersed cells of *Halomonas* growing in alginate beads which produces poly-β-hydroxyalkanoates (PHA), accumulate higher concentrations of these compounds than their counterparts growing planktonically, in the same unbalanced stress-inducing culture medium [69]. As established cell factories, biofilm-detached cells could be a better alternative for PHA production than planktonic cells.

## Biofilms in food: the case study of table olives

Olives are a fruit that contains a bitter component (oleuropein), sugar (2.6–6.0 %), and high oil content (12–30 %), in proportions that change according to the degree of maturity and variety of the olive. Because olives are not palatable directly after their harvest, they must be treated before they can be sold to consumers. Treatments to obtain table olives include alkaline processing of green olives (“Spanish style”), alkaline oxidation of ripe olives (“Californian style”), and direct immersion in brine (“natural olives”) [80]. All of these treatments include immersion of the olives in brine (7–10 % NaCl solution) that is gradually enriched with nutrients from the olive mesocarp, which serves as the substrate for microorganisms to initiate fermentation. The final product has improved sensory characteristics and can then be marketed [81].

Fermentation depends on the activities of a mixed community of microorganisms that form a biofilm on the skin of the olives [80]. Depending on the initial conditions, lactic acid bacteria (LAB) grow spontaneously on treated olives, although they can be substituted by yeasts, such as *Candida*, *Pichia*, and *Saccharomyces* [80, 82], in truly natural olives. The main microbial genus isolated from table olives is *Lactobacillus*. Other, albeit quantitatively less important genera of LAB isolated from olives are *Enterococcus*, *Pediococcus*, *Leuconostoc*, and *Lactococcus* [81]. LAB metabolizes sugars, mainly converting them to lactic acid. They play key roles in the preservation of many foods and contribute to improving sensory properties such as texture and flavor [83].

During the fermentation of “Spanish-style” green olives, LAB and yeasts form an authentic biofilm with an exopolysaccharide matrix [84, 85]. In a scanning electron microscopy study, Grounta and Panagou [86] showed the formation of biofilms on oxidized Greek black olives, while Benítez-Cabello et al. [80] described the formation of microbial biofilms on the epidermis of directly brined “natural” green olives.

The fermentation ecosystem of truly natural olives, like that of treated olives, consists of a complex mixture of gram-negative bacteria, LAB, and yeasts [87]. Gram-negative bacteria are very important during the initial phase of the process and reach a maximum abundance on the second day after the olives have been placed in brine. Thereafter, the abundances of LAB or yeasts, or both, depending on the nature of the fermentation, gradually increase, replacing the gram-negative bacteria and consuming the nutrients in the medium while excreting lactic or citric acids and volatile compounds [81, 87]. Among the latter, the most abundant is ethanol, followed by methanol, whereas propanol, 2-butanol, ethyl acetate, acetaldehyde, and dimethyl sulfide are detected in lower amounts [88]. Ethanol is the precursor of ethyl esters, with ethyl acetate as the most important with respect to the palatability of olives. Both propanol, which originates mainly from yeast metabolism, and ethanol contribute to the secondary odor of fermented olives [89]. Thus, regardless of the olive processing method, biofilm-forming microorganisms determine the quality, safety, flavor, and taste of the final product. Other examples of popular foods that depend on the microbial activities of complex biofilms on the surface of the fruit are cocoa [90] and coffee [91].

## Looking into the future: designing synthetic microbial communities for biotechnological processes

The metabolic capabilities of microorganisms have been the basis for many major biotechnological advances, most of which have made use of genetically modified single microbial strains [92, 93]. Newly acquired properties and therefore the biosynthesis of compounds of interest may depend on interference with innate metabolism, genetic instability, and the production of undesirable side-products [93, 94]. However, the number of new properties that can be incorporated into a single microorganism is limited [92]. In natural communities, microbial populations interact constantly with each other, establishing complex ecological webs [95]. The wide-ranging metabolic plasticity of microbial communities includes the efficient catalysis of many processes, by combining the metabolic pathways and enzymatic systems of the different resident organisms [24, 96].

Synthetic biology is an emerging field of research in which engineering strategies are used to program biological systems. It is based on the systematic characterization of the genetic and metabolic pathways present in microbial consortia and an understanding of the molecular mechanisms underlying their interactions. In the design of engineered bacterial consortia, three fundamental aspects must be taken into account: (i) the type of ecological interaction (e.g., commensalism, mutualism, competition, predation, or parasitism) to be established [92, 93, 96]; and (ii) the interactions between two or more microbial strains that are needed to stabilize and optimize the synthetic consortia for bioprocessing applications [94, 97–99]. Stabilization and functionality may be related distribution of microbial strains inside the biofilm [98, 99].

Cross-feeding is a common element in commensalistic and mutualistic interactions [93]. In a commensalistic interaction, organism A benefits from the interaction, for example, by scavenging several products released by organism B, which is neither positively nor negatively affected by this activity. In a mutualistic interaction, organisms A and B benefit, for example, by exchanging metabolites or by removing one another’s inhibitory substances. Axenic cultivation disrupts these mutualistic interactions and may be one of the reasons for the low cultivation efficiency of environmental microorganisms [93]. By contrast, cultivation strategies that preserve microbial interactions have led to much higher cultivation efficiencies [100, 101]. However, microorganisms also compete with their neighbors for space and resources. In competition, as in predator–prey or parasitic interactions, only one partner benefits, while the other is adversely affected. Competition is related to the rapid uptake of nutrients and conversion into biomass for one member of the competing populations, with the balance depending on the environmental conditions. Amensalism is a special type of competitive interaction between two populations in which one population adversely affects the other without being affected itself. For example, population A can excrete several compounds into the surrounding environment, such as acids, alcohols, bacteriocins and antibiotics, that are effective inhibitors of the growth of population B [102].

A challenge in synthetic biology is to coordinate several populations to produce a value-added product at higher yield [94, 99]. Consortia are frequently unstable basically because co-evolution of constituent members may affect their interactions, which are the basis of collective metabolic functionality. This realization shows the necessity to better identify the key components that influence the stable coexistence of microorganisms. Christian et al. [103] used the KEGG database and extracted 447 organism-specific metabolic-networks and then performed pairwise comparisons by integrating metabolic networks. The work simulates the metabolic cooperation of two organisms and at different conditions to prove their stability and efficiency. Others authors also modeled other ecosystem based also on metabolic networks [96, 104]. But, once an artificial community has been created or modelled, it needs to be combined with experimental work. Such models can then be further refined through experimental data by modifying the parameters of the system for the optimum functioning. Different media formulations (based on carbon, nitrogen, sulfur and phosphorus) can affect how two microbes will ultimately interact [105]. Microorganisms can even be genetically manipulated to achieve new interaction types [106, 107].

Several synthetic communities have been used for the synthesis of different products in the industry. Industrially ethanol biosynthesis is produced by the fermentation of glucose or sucrose from sugar cane or beets. But alternative sources of sugar have been investigated, such as lignocellulosic substrates. Co-cultures of of *Zymomonas mobilis* and *Candida tropicalis* were able to transform enzymatically hydrolyzed lignocellulosic biomass into ethanol with a yield of 97.7 % [108]. Synthetic microbial communities consisting of *Ketogulonicigenium vulgare* and *Bacillus megaterium* have been used in industry to produce 2-keto-gulonic acid (2-KGA), the precursor of vitamin C [109]. In this process, *K. vulgare* is the 2-KGA producing strain and *B. megaterium* acts as a companion strain that secretes some metabolites (such as l-glycine, l-proline, l-threonine, and l-isoleucine) to stimulate the growth of *K. vulgare* and, thus, to enhance 2-KGA production [110].

The success achieved thus far with synthetic microbial communities has demonstrated that genetic circuitries can be engineered to construct efficient cellular factories [92]. Xia et al. [111] described a consortium of *Escherichia coli* strains that could simultaneously utilize glucose, xylose, arabinose, and acetate. However, the efficient microbial utilization of lignocellulosic hydrolysates has remained challenging because in addition to multiple sugars lignocellulose contains growth inhibitors, such as acetic acid (or acetate). Nonetheless, in an engineered yeast consortium the utilization of cellulosic substrates was exploited to increase the production of ethanol [112].

We conclude this review by emphasizing the importance of detailed studies of biofilm ecology, such as their populations, the interactions among these populations, their functionality, and community and ECM structure, to achieve a complete understanding of microbial systems. This, in turn, will allow their successful application to obtain value-added products. Indeed, a biofilm is much more than the sum of its integrative parts.
